# HSP70 and Primary Arterial Hypertension

**DOI:** 10.3390/biom13020272

**Published:** 2023-02-01

**Authors:** Bernardo Rodriguez-Iturbe, Richard J. Johnson, Laura Gabriela Sanchez-Lozada, Hector Pons

**Affiliations:** 1 Department of Nephrology, Instituto Nacional de Ciencias Médicas y Nutrición “Salvador Zubirán”, Mexico City 14080, Mexico; 2 Division of Renal Diseases and Hypertension, University of Colorado Anschutz Medical Campus, Aurora, CO 80045, USA; 3 Department of Cardio-Renal Physiopathology, National Institute of Cardiology Ignacio Chávez, Mexico City 14080, Mexico; 4 Facultad de Medicina, Universidad del Zulia, Maracaibo 4011, Venezuela

**Keywords:** HSP70, hypertension, immune reactivity, inflammation

## Abstract

Heat shock protein 70 (HSP70) production is a stress-generated cellular response with high interspecies homology. HSP70 has both chaperone and cytokine functions and may induce, depending on the context, tolerogenic anti-inflammatory reactivity or immunogenic and autoimmune reactivity. Intracellular (chaperoning transit of antigens to MHC in antigen-presenting cells) and extracellular HSP70-related effects are associated with hypertension, which is an inflammatory condition recognized as the most important risk factor for cardiovascular disease mortality. Here, we review (a) the relationship between HSP70, inflammation and immune reactivity, (b) clinical evidence relating to stress, HSP70 and anti-HSP70 reactivity with primary hypertension and (c) experimental data showing that salt-sensitive hypertension is associated with delayed hypersensitivity to HSP70. This is a consequence of anti-HSP70 reactivity in the kidneys and may be prevented and corrected by the T-cell-driven inhibition of kidney inflammation triggered by specific epitopes of HSP70. Finally, we discuss our postulate that lifelong stress signals and danger-associated molecular patterns stimulate HSP-70 and individual genetic and epigenetic characteristics determine whether the HSP70 response would drive inflammatory immune reactivity causing hypertension or, alternatively, would drive immunomodulatory responses that protect against hypertension.

## 1. Introduction

Heat shock proteins (HSPs) are evolutionarily preserved immunodominant proteins that are constitutively expressed and rapidly increase to represent as much as 15% of cellular protein content in response to stressful stimuli [1]. Stress-induced overexpression aims to prevent the cellular aggregation of misfolded proteins by chaperoning their refolding or their degradation if they are irreparably damaged [2]. The adaptive reactivity generated by HSP activation includes antioxidant responses that prevent oxidative stress, suppress inflammation and inhibit apoptosis by binding proapoptotic factors and blocking Bax mitochondrial translocation [3]. The overall results of these actions are the protection of biological pathways critical for cell function and the prevention of DNA fragmentation [4].

In addition to their cytoprotective functions, HSPs are immunodominant molecules that are important in the recognition of infectious agents and drive antigens to the mixed histocompatibility complex (MHC) in antigen-presenting cells (APC) [5]. Hence, HSP-driven modifications in the course of diseases depend on the specific cell type and the pathogenic dysregulation involved. HSP responses inducing anti-inflammatory cytokine responses and regulatory T-cell expression are beneficial to downregulate uncontrolled autoimmune reactivity, but the suppression of inflammation may be deleterious when inflammation constitutes a defense mechanism. For instance, HSP expression in synovial tissues is anti-inflammatory and protective in rheumatoid arthritis [6], while the stimulation of antigen-presenting cells by HSP70 may be responsible for acute asthmatic attacks [7] and the HSP70-mediated suppression of immune reactivity and apoptosis may represent a survival factor for cancer cells [8].

## 2. HSP70, Inflammation and Immune Reactivity

HSP proteins have a highly preserved interspecies homology and molecular mimicry makes self-HSPs potential targets for disease-related T-cell responses. HSP70 is the most extensively studied HSP and in the human is comprised of a family of 13 members located in cytosol, nuclei, mitochondria, and endoplasmic reticula. The structure of HSP70 family members includes an N-terminal nucleotide-binding domain, a C-terminal substrate-binding domain and a C-terminal tail that binds with co-chaperones. Functionally, HSP70 proteins are characterized by substrate recognition, adaptive responses imposed by cellular and extracellular localizations, and interactions with other chaperones that control the presentation of client proteins to HSP70 and their refolding after HSP70 activity [9,10].

HspA1A and HspA1B are the major stress-inducible HSP70 proteins of the HSP70 family. HSPs are intracellular proteins and extracellular locations of HSP70 (complexed with antigenic peptides, in exosomes and in free soluble form) may be a consequence of cell necrosis or result from release in exosomes, extrusion in cholesterol microdomains or lysosomal secretion [10]. 

HSP70 proteins induce intricate and contrasting immunomodulatory responses that drive the development of anti-inflammatory or proinflammatory immune reactivity. Collaborations between HSP70 and other co-chaperones, most notably HSP90 and HSP40 [11,12,13], are part of the HSP70 chaperone reaction cycle [10] and constitute a central characteristic of the heat shock response (HSR) that contributes to its pleiomorphic manifestations. HSP70 and HSP40 share a J-domain, DnaJ, necessary but not sufficient for the ATPase activity. A HSP40–HSP70 partnership is required for ATP hydrolysis and for substrate identification and, therefore, it is essential for HSP70-induced protein disaggregation, which is a preliminary step for subsequent refolding. The collaboration between HSP70 and HSP40 in the protection of proteins has been extensively reviewed [11]. HSP90 is another co-chaperone that has a large diversity of substrates and acts downstream of HSP70. While HSP70 binds to short hydrophobic segments, HSP90 interacts with extended binding domains of 50 to 100 aminoacids, driving the folding of proteins that restores their native condition. The absence of HSP90 results in the degradation of many substrates, most notably the receptors of steroid hormones. Interactions between HSP70 and HSP90 have been comprehensively reviewed [12,13].

**Figure 1:**
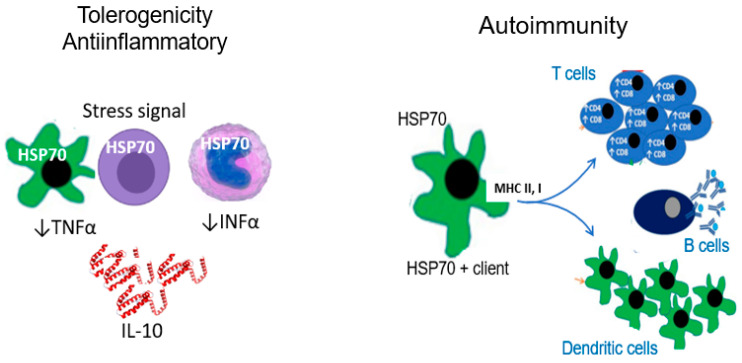
Immune reactivity induced by HSP70. HSP70 response to stress may induce tolerogenic anti-inflammatory responses as well as immune reactivity. Intracellular HSP70 activation suppresses NLRP3 inflammasome activation. In addition, specific polypeptide sequences of HSP70 induce a tolerogenic responses characterized by IL-10 immunomodulatory secretion. In contrast, extracellular HSP70 induce immune reactivity that includes anti-HSP70 antibody formation. The chaperoning function of HSP70 include the protection and transfer of exogenous and endogenous antigenic peptides to the MHC I and II and the subsequent adaptive immune reactivity. Tolerogenic responses to intracellular HSP70 in dendritic cells and macrophages include reduction of TNFα and INFα and production of IL-10 by T cells. Suppression of T cell-induced inflammatory responses is independent of its effects on monocyte-derived immature dendritic cells [17].

The immune reactivity induced by HSP70 depends, at least in part, on the location of HSP70 activity: intracellular HSP70 is anti-inflammatory, while extracellular HSP70 may induce anti-inflammatory and proinflammatory responses [14,15,16] (Figure 1).

Martine et al. [18] have shown that HSP70 is a negative regulator of NLRP3 inflammasome in vivo and in vitro. Their studies focused on inducible HSP70 and showed that HSP70 deficiency enhances caspase -1 activation and IL-1β production in activated macrophages and overexpression of HSP70 and heat shock inhibits NLRP3 inflammasome activation and reduces caspase and IL-1β production.

Tolerogenic responses are not exclusive of intracellular HSP70 and may also be induced by extracellular HSP70 delivered to or present in cellular membranes or in ectosomes [19]. In vitro treatment of peripheral blood mononuclear cells of patients with rheumatoid arthritis with HSP70 induce the production of IL-10 and *Mycobacterium tuberculosis* HSP70 (mtHS70) blocks the maturation of bone marrow precursors that fail to express MHC class II and CD86. Using an innovative method of immersing graft tissue with mtHSP70, Borges et al. [20] showed that soluble human HSP70 induces a regulatory phenotype in monocyte-derived dendritic cells and inhibits acute allograph rejection. The internalization of extracellular HSP70 likely depends on scavenger receptors, such as LOX-1 (lectin-like oxidized low-density lipoprotein receptor 1), SREC-1 (scavenger receptor-1) and FEEl-1 (scavenger receptor expressed by endothelial cells-1). Intracellular signaling is apparently mediated by the activation of MAPK/ERK (mitogen-activated protein kinase / extracellular signal-regulated kinase) and STAT3 (Signal transducer and activator of transcription 3) pathways that stimulate Il-10 production and tolerance [19]. The clinical significance of the immune suppressive response that HSPs may induce was shown in seminal studies conducted by van Eden et al. [21,22,23]. Van Eden et al. showed that *Mycobacterium TBC* contained an epitope that was cross-reactive with self-antigens present in the joint cartilage of rats with adjuvant arthritis and in T lymphocytes of patients with rheumatoid arthritis. Screening mycobacteria antigens showed that T-cell clones of adjuvant arthritis recognized the aminoacid sequence in position 180–188 of *Mycobacterium bovis* BCG antigen and further showed that administration of this antigen induced resistance to adjuvant arthritis in rats [21]. In subsequent studies, the authors identified specific peptide sequences of mycobacterial HSP that stimulated a protective self-HSP cross-reactivity, inducing IL-10 mediated suppression of autoimmune colitis and autoimmune encephalitis [22,23].

Tolerogenic responses are not exclusive of intracellular HSP70 and may also be induced by extracellular HSP70 delivered to or present in cellular membranes or in ectosomes (reviewed in [19]). The in vitro treatment of peripheral blood mononuclear cells of patients with rheumatoid arthritis with HSP70 induces the production of IL-10, and *Mycobacterium tuberculosis* HSP70 (mtHS70) blocks the maturation of bone marrow precursors that fail to express MHC class II and CD86. Soluble human HSP70 induces a regulatory phenotype in monocyte-derived dendritic cells and soluble mtHSP70 inhibits acute allograph rejection [20]. The internalization of extracellular HSP70 likely depends on scavenger receptors, and signaling is transmitted through the TLR2 and ERK pathways [19]. The clinical significance of the immune-suppressive response that HSPs may induce was shown in seminal studies by van Eden et al. [21,22,23], who used specific peptide sequences of mycobacterial HSP to stimulate protective self-HSP cross-reactivity to induce the IL-10-mediated suppression of adjuvant arthritis, colitis and autoimmune encephalitis.

Proinflammatory effects of HSP70 were studied by Asea et al. [24], who demonstrated that the treatment of monocytes with exogenous HSP70 resulted in the phosphorylation of inhibitory I-κBα subunit at Ser3 and the activation of N*F*kB. HSP70 activates monocytes by inducing intracellular Ca++ flux and additional pathways, independent and dependent of CD14, that generate the release of tumor necrosis factor (TNF)-α, interleukin (IL)-1β and IL-6. Some studies of the proinflammatory stimulation induced by HSP70 were controversial and attributed to contamination with endotoxin [25], but the extracellular expression of HSP70 and the existence of anti-HSP70 serum antibodies are beyond dispute, as is the role that HSP70 plays in acquired immunity chaperoning intracellular and extracellular antigens to MHC I and II in APC in canonical and cross-presentation path (reviewed in [26]).

The potential of HSP70 to induce autoimmunity in vivo was demonstrated by Millar et al. [27] in the model of autoimmune diabetes. Their studies showed that the tolerance induced by the immunodominant gp33 epitope was changed to a rapid autoimmunity response, resulting in diabetes with the administration of HSP70. Furthermore, HSP70 may also determine the selection of the epitope chosen for the presentation of autoreactive T cells [28]. In human autoimmunity diseases, the existence of anti-HSP70 antibodies is well-recognized (reviewed in [29]).

A major insight into the contrasting proinflammatory and anti-inflammatory mechanisms activated by HSP70 was obtained through the studies of Fong et al. [30], who investigated the relationship between HSP70 and Ig-related lectins that recognize sialic acid (Siglecs). Sialic acids are self-associated molecular patterns (“SAMP”) essential to self-recognition by the immune system and Siglec-5 and Siglec-14 are paired receptors that recognize identical ligands but send opposing immune reactivity signals. Siglec-5 and Siglec-14 were identified as ligands of HSP70, and their response was anti-inflammatory and proinflammatory, respectively, suggesting a likely explanation for the contradictory results reported in the extracellular HSP70 activation [30].

Of relevance to the relation between HSP70 and blood pressure is the recognition that stress-induced HSP70 response may suppress as well as stimulate inflammation. The contrasting HSP70 response could generate and aggravate or prevent and ameliorate hypertensive cardiovascular disease.

## 3. HSP70 and High Blood Pressure

Hypertension is an inflammatory condition in which immune reactivity, innate and acquired, plays a critical pathogenic role [31,32,33,34,35]. Autoimmune reactivity triggered by protein adducts generated by isoketals (gamma ketoaldehydes) resulting from lipid peroxidation has been reported in experimental models of hypertension and plasma F2-isoprostanes, which are formed in concert with isoketals, were found to be elevated in [36]. Our group has suggested that HSP70 is a critical element in the pathogenesis of hypertension by either generating anti-HSP70 immune reactivity and/or driving autoantigens to the MHC in antigen-presenting cells [26,31,37].

Hypertension has been mechanistically associated with inflammation resulting from innate and acquired autoimmunity originating from danger-associated molecular patterns (DAMPs) generated by metabolic stress. Metabolic stress during a life span is impossible to determine, but is likely comparable in the vast majority of hypertensive and normotensive individuals. We suggest that the individual HSP70 response to stress, proinflammatory or anti-inflammatory, is the determining factor in the development of or protection from hypertension.

## 4. Stress, HSP70 and Hypertension

The association of environmental stress with an increase in blood pressure was reported more than 60 years ago in studies that showed that, in the general population, blood pressure was higher during the winter [38]. This finding was attributed to increased sympathetic activity in cold weather. However, the seasonal variability of blood pressure, while evident in hypertensive patients, was not confirmed in normotensive individuals, and changes in plasma and urinary catecholamines in the winter were similar in normotensives and hypertensives [39]. Comprehensive studies by the group of Pavel Hamet in Montreal confirmed an inverse relationship between blood pressure and daily temperature in hypertensive patients [40], and subsequent investigations in experimental models of hypertension demonstrated the increased sensitivity of blood pressure to various stresses. For instance, McMurthy et al. [41] showed that exposure to 50 °C for 1 min or to ether vapors raised blood pressure in spontaneously hypertensive rats (SHRs) more than in normotensive rats. The hypertensive response was preceded by hormonal changes, particularly in aldosterone and corticosterone blood levels.

Recognizing that heat shock protein activation is an evolution-preserved response to stress, several investigations showed that imposing thermal, painful and restrained conditions induced exaggerated increments in HSP70 mRNA in SHRs, hypertensive mice strains [42] and hypertensive humans [43]. These studies prompted the hypothesis that genetic characteristics in hypertensives could be responsible for the overexpression of HSP70 due to environmental stress. This hypothesis was supported by studies that localized the HSP70 gene near the MHC in SHRs [44].

While stressful stimuli induce an overexpression in HSP70 mRNA in hypertensive rodents and hypertensive humans, this exaggerated response is transient and may be a protective response. Chen et al. [45] showed that raising the body temperature to 42 °C for 45 min a day before the administration of angiotensin II suppressed the expression of NF*k*B in the aorta and reduced hypertension.

The increase in blood pressure by itself is a stress factor that stimulates HSP70. Xu et al. [46] showed that phenylephrine, dopamine, vasopressin, angiotensin II, and endothelin-1 increased HSP70 mRNA in cells harvested from the aorta 60 min after a blood pressure increase. Furthermore, they found a correlation between the increase in blood pressure and HSP70 expression and suggested that the findings indicated a protective response against hypertension.

Significantly, the immediate and short-term HSP70 increment resulting from stress differs from the lifelong increment in blood pressure characteristic of hypertensive cardiovascular disease. In fact, quite the opposite is suggested by studies that show that the same stress, if applied repeatedly, reduces baseline blood pressure. Kvetnansky et al. [47] induced repeated restrain episodes in SHRs and found that, contrary to expectations, the acute increment in blood pressure observed initially was attenuated, and the baseline blood pressure was normalized despite increased baseline levels of norepinephrine. Similar results were reported by Malo et al. [48], who showed that daily exposure to 50 °C for 5 min progressively lowered the basal blood pressure of hypertensive mice to levels similar to that of normotensive mice. This phenomenon was associated with a faster declination of HSP70 mRNA and protein and is relevant to reinforce that repeated episodic stress, by itself, does not play a role in chronic increments in blood pressure.

## 5. Anti-HSP70 Antibodies in Primary Hypertension

Table 1 summarizes the studies that showed an association between hypertension and HSP70 expression [31,43,49,50,51,52,53,54,55,56,57,58,59].

Evidence against the possibility that HSP70 overexpression is a protective response to hypertension was obtained in young prehypertensive SHRs. The overexpression of HSP72 was observed in 4-week-old SHRs, anteceding the development of hypertension [31]. Wu et al. [50] studied 764 steel mill workers from 6 work sites and showed that harsh working conditions (noise, dust and heat) were associated with an incidence of anti-HSP70 antibodies in 40.6% of hypertensive patients and only 18.6% of normotensive individuals; furthermore, statistical correlations between anti-HSP70 levels and hypertension were more significant in severely (>160/95 mmHg) hypertensive patients. Studies of circulating lymphocytes showed that lymphocytes harvested from hypertensive patients have a more intense HSP70mRNA expression than lymphocytes of normotensive individuals [43]. Bravo et al. [49] showed that HSP70 and vimentin are overexpressed in the cortical proximal tubular cells of the kidney in experimental modes of salt-sensitive hypertension. The constitutively expressed levels of HSP70 and their relationship with inflammatory markers were studied by Srivastava et al. [51] in 132 hypertensives and 132 normotensive individuals; they found that HSP70 mRNA in whole blood and HSP70 expression in plasma were increased in hypertensive patients. In addition, IL-6 and TNFα in hypertensive patients, but not in controls, were significantly correlated with HSP70 gene expression. Pockley et al. [52] studied serum samples obtained in the European Lacidipine Study on Atherosclerosis (ELSA) and the Swedish population-screening program from 111 hypertensive patients and 75 normotensive controls and found increased levels of antiHSP70 and anti-HSP65 antibodies in hypertensive patients. Similar results were found by our group [53]. Pons et al. [59] found that peripheral blood lymphocytes of patients with primary hypertension, in contrast to lymphocytes of normotensive individuals, develop a proliferative response when challenged with a specific HSP70 peptide sequence.

Genetic studies were done by Li et al. [54] focusing on polymorphisms of the HSP70 family and the risk of hypertension in the Uygur population in China that have a high homogeneity and unique life styles. HSP70 polymorphisms were associated with 3 to 5-fold increased risk of hypertension. Zheng et al. [55] also focused in HSP70 polymorphisms in oven workers in China and found a 2 to 4-fold increased relative risk with hypertension.

## 6. Hypertension Is Induced by Immune Reactivity to HSP70

**Figure 2:**
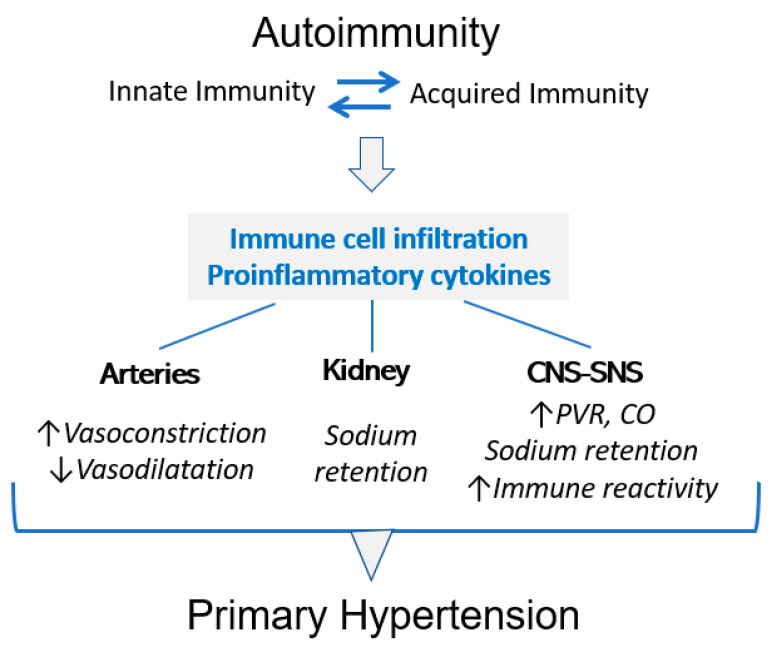
Autoimmunity in primary hypertension. Autoimmune reactivity is a central characteristic of primary hypertension. Prohypertensive immune reactivity has been demonstrated in the arterial adventitial layers, in the tubulointerstitial areas of the kidney and in central and sympathetic nervous systems. PVR: éripheral vascular resistance; CO: cardiac output.

The association between immune reactivity and hypertension is well-established. A large number of studies in genetic and induced experimental hypertension have demonstrated that hypertension is corrected or prevented by immunosuppressive anti-inflammatory treatment (reviewed in [31,37]). Autoimmune reactivity induces hypertension that, as a consequence of inflammatory infiltration in the adventitia of arteries and tubulointerstitial areas of the kidney, increases sympathetic nervous system activity (Figure 2). Barhoumi et al. [56], studying angiotensin-induced hypertension, showed that macrophage and T-cell infiltration of the aorta were prevented by the adoptive transfer of T-reg cells, which ameliorated hypertension. The activation of sodium reabsorption in the kidney induced by immune reactivity was demonstrated by Norlander et al. [57], who found that experimental hypertension was associated with T-cell production of IL-17 and that hypertension was blunted in the IL-17 ^-/-^ mice. IL-17 prohypertensive effects include the activation of sodium chloride co-transporter and epithelial sodium channel in the distal tubule. Increase sympathetic activity not only results in vasoconstriction and increased sodium reabsorption, but stimulates immune cell release from the spleen and supports immune responses [31,58].

The role played by the HSP70 response in hypertension was investigated in the experimental model of salt-sensitive hypertension (SSHTN) induced by the transient (3-week) administration of N^w^-nitro-L-arginine methyl ester (LNAME) [60]. In these studies, we used a specific amino acid sequence of *Mycobacterium tuberculosis* HSP70 (mtHSP70) shown by Wendling et al. [61] and Praken et al. [62]) to be immunogenic and to induce the generation of T cells that were cross-reactive with the homologous rat HSP70 sequence. In their experiments [60,61], these peptide-induced T cells and T cells generated by the whole HSP70 produced IL-10 and, if administered intranasally, inhibited adjuvant arthritis. Rats with SSHTN were shown to have delayed hypersensitivity to mtHSP70 and their splenocytes had a clonal CD4 response when challenged with mtHSP70.

**Figure 3:**
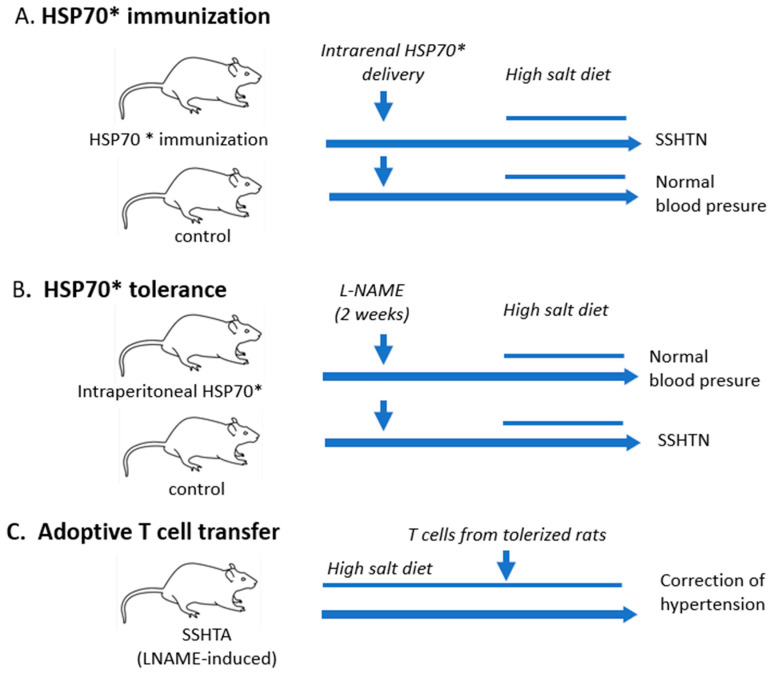
HSP70 immune reactivity and hypertension. (**A**) Immunization with a specific amino acid sequence * (59) of *Mycobacterium tuberculosis* HSP70 in the foot pads followed by intrarenal plasmid-delivered administration of HSP70 results in tubulointerstitial inflammation and salt-sensitive hypertension (SSHTN). (**B**) The induction of immune tolerance with the intraperitoneal administration of the antigenic sequence used to induce immune reactivity results in prtection against SSHTN. (**C**) Adoptive transfer of T cells obtained from rats tolerized to HSP70 corrects hypertension. (Data from Reference [59]).

Immunization in the foot pads with mtHSP70 followed by the expression of HSP70 induced in the kidney via plasmid injection generated a tubulointerstitial inflammatory infiltrate of macrophages and lymphocytes and the development of SSHTN [59] (Figure 3A).

## 7. Immune Tolerance to HSP70 Corrects Hypertension

The participation of immune reactivity to HSP70 in the pathogenesis of SSHTN was further investigated by the induction of immune tolerance to HSP70 by the intraperitoneal administration of mtHSP70. The effectiveness of tolerization was confirmed by the suppression of delayed hypersensitivity to HSP70, the induction of T regulatory cells, the reduction in proinflammatory cytokines (IL-6) and the increase in IL-10 in the kidneys of rats with SSHTN. Furthermore, tolerization resulted in the inhibition of tubulointerstitial inflammation, which is the hallmark of SSHTN. Immune tolerance to HSP70 prevented the development of SSHTN (Figure 3B). In addition, the adoptive transfer of T cells from tolerized rats to rats with SSHTN corrected hypertension (Figure 3C) [59].

## 8. HSP70-Induced Immune Reactivity Modulates Inflammation in Hypertension

We contend that the characteristics of HSP70-induced immune reactivity are a central determinant of blood pressure. Overall biological stress is likely comparable in the majority of hypertensive and normotensive individuals, and increased or normal blood pressure is not a consequence of stress signals but instead the result of the proinflammatory or anti-inflammatory HSP70 response to those signals. It should be recognized that hypertension is a quantitative, rather than qualitative, deviation of the norm, and the division between normal and increased blood pressure is consensual and based on the incidence of associated complications [63,64]. As discussed earlier, stress-activated HSP70 overexpression may result in the stimulation or suppression of inflammation that, when present in the kidneys, the arterial walls and the central and sympathetic nervous system, determines primary hypertension.

In addition to stress-related HSP70 activity, autoimmunity may be induced by extracellular HSP70 antigenic determinants [16]; furthermore, HSP70 may play a supporting role in the antigenicity of other antigens identified in hypertensive patients [36].

**Figure 4:**
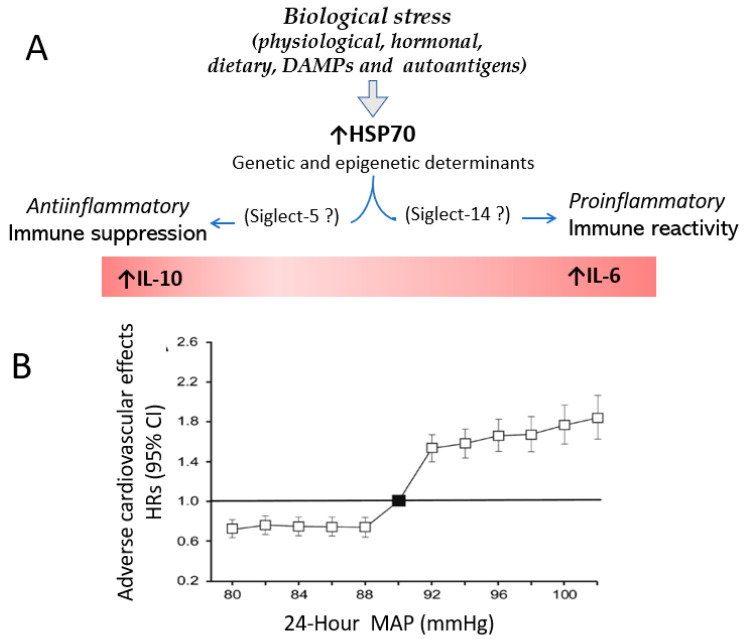
Stress-induced HSP70 overexpression and blood pressure. (**A**) We posit that biological stress induces overexpression of HSP70 that, depending on genetic and epigenetic determinants, induces or suppresses immune reactivity that drives or prevents hypertension. Adverse effects of hypertension are associated with inflammation. The potential role of interaction between HSP70 and Siglec-5 and Siglec-14 is discussed in the text. (**B**) Increased incidence of fatal and non-fatal cardiovascular outcomes is associated with 24 h mean arterial pressure of 92 mmHg or higher (Figure 4B was published as Figure 1A in the open access paper of Melgarejo JD et al. [66]).

As shown in Figure 4, we posit that the immune reactivity resulting from HSP70 stimulation is responsible for the prevalence of high blood pressure (>130/80 mmHg) in 51% of males and 39.7% of females 18 years of age and older in the United States [65].

In contrast with the stimulation of both innate and adaptive immunity in patients that develop severe hypertension, the remaining normotensive population responds to metabolic stress with a protective downregulation of inflammatory reactivity, which, as discussed earlier, is also a recognized HSP70 response to stress. The identification of genetic and epigenetic characteristics that determine the specific type of HSP70 response [67] and, perhaps, the differential engagement of Siglec-5 and Siglec-14, is critical in developing strategies for controlling hypertension.

## 9. Conclusions

There is considerable clinical evidence that associates stress, HSP70 and primary hypertension. Experimental data indicate that tubulointerstitial inflammation of the kidneys and salt-sensitive hypertension may be driven by immune reactivity to HSP70 and may be prevented and corrected by T-cell immunoregulatory responses that HSP70 may drive. The data on hypertension resemble the findings regarding other autoimmune diseases such as adjuvant arthritis in which disease activity is modulated by the induction of autoreactive HSPs [23]. We suggest that genetic and epigenetic factors determine whether HSP70-driven autoimmune reactivity results in prohypertensive inflammation or a protective anti-inflammatory response that prevents the development of hypertensive cardiovascular disease. The possibility of intervening in the HSP70 response should be considered a potential therapeutic strategy in primary hypertension.

## Figures and Tables

**Table 1:** Association between HSP70 and hypertension.

Study	Experimental/Clinical	Results	References
HSP70 expression	Salt-sensitive hypertension	Overexpression in proximal tubular cells of the kidney	[49]
HSP70 expression in relation to development of hypertension	Spontaneously hypertensive rats	HSP72 overexpression precedes hypertension	[31]
Working conditions and anti-HSP antibodies	Harsh working conditions	Anti-HSP70 antibodies in 40.2% of hypertensives and 18.6% of normotensive individuals	[50]
Heat-induced HSP70 mRNA in lymphocytes	15 min at 42 °C in peripheral blood lymphocytes	Increased HSP70 mRNA in lymphocytes from hypertensive patients	[43]
Constitutive HSP70 mRNA and expression and inflammation	132 hypertensives and 132 normotensive individuals	6-fold increase in HSP70 mRNA in hypertensives. Positive correlation with IL6 and TNFα levels	[51]
Constitutive anti-HSP antibodies	Serum samples from European Lacidipine Study on Atherosclerosis (ELSA) and from Swedish population-screening programme	Anti-HSP70 and anti-HSP65 levels increased in hypertensive patients	[52,53]
Peripheral blood lymphocytes challenged with a mycobacterial HSP70 peptide sequence	10 patients with primary hypertension, 12 normotensive patients	Proliferative response (x2 proliferation index) in patients with primary hypertension	[59]
Genetic HSP70 polymorphisms	Uygur ethnicity	5 polymorphisms of HSP70 associated with increased risk of hypertension	[54]
HSP70 polymorphisms and working conditions	Coke oven workers	Increased hypertension risk: HSP70-1 GC, CC and C+CCgenotype Reduced hypertension risk: HSP70-2 AG, GG and AG+GGgenotype	[55]

## Data Availability

Not applicable.
